# Formulating a Stable Mannitol Infusion while Maintaining Hyperosmolarity

**DOI:** 10.3390/pharmaceutics12020187

**Published:** 2020-02-21

**Authors:** Oisín Kavanagh, Fiona Hogan, Caoimhe Murphy, Denise Croker, Gavin Walker

**Affiliations:** Solid State Pharmaceutical Centre (SSPC), The Science Foundation Ireland Research Centre for Pharmaceuticals, Department of Chemical Sciences, Bernal Institute, University of Limerick, V94 T9PX Limerick, Ireland; 116307026@umail.ucc.ie (F.H.); 116396751@umail.ucc.ie (C.M.); Denise.Croker@ul.ie (D.C.); Gavin.Walker@ul.ie (G.W.)

**Keywords:** mannitol instability, raised intracranial pressure, intracranial hypertension, traumatic brain injury, reformulation, mannitol, recrystallisation

## Abstract

Mannitol infusion is commonly used in the treatment of intracranial hypertension following traumatic brain injury. It has long been known to have stability issues, specifically, mannitol recrystallises from solutions greater than 10% *w*/*v* in ambient conditions. This can happen at any time, whether on the pharmacy shelf or during a medical procedure. This study describes the stability limits of 20% *w*/*v* mannitol infusion (the most common strength used clinically) and proposes a number of safer, stable and tuneable hyperosmotic formulations of mannitol in combination with clinically acceptable osmotic agents (NaCl, sorbitol and glycerol).

## 1. Introduction

Traumatic brain injury (TBI) is one of the greatest causes of death and disability in those aged under 40 [1], and with 69 million cases of TBI each year [2], this presents a critical health problem. The pathological mechanism behind this death and disability (besides the immediate trauma) is cerebral oedema, which elevates intracranial pressure, and can result in cerebral ischaemia [3]; as such, intracranial hypertension is a medical emergency.

As increases in intracranial pressure (ICP) are inversely proportional to survival [4], treatment aims to reduce ICP <20 mmHg as soon as possible [5]. Commonly, intravenous mannitol or hypertonic saline infusions are employed. There are two mechanisms which contribute to their effect: (1) creation of an osmotic gradient, which draws cerebral fluid across the blood–brain barrier and into the plasma and, (2) plasma expansion with associated reductions in blood haematocrit and viscosity [6]. Although there is significant debate regarding the choice of hyperosmolar therapy [7,8,9,10]—highlighted by the recent Sugar or Salt trial [11]—ultimately, the choice of hyperosmolar agent is a complex decision [5], and each treatment option carries its own advantages and disadvantages.

**Figure 1:**
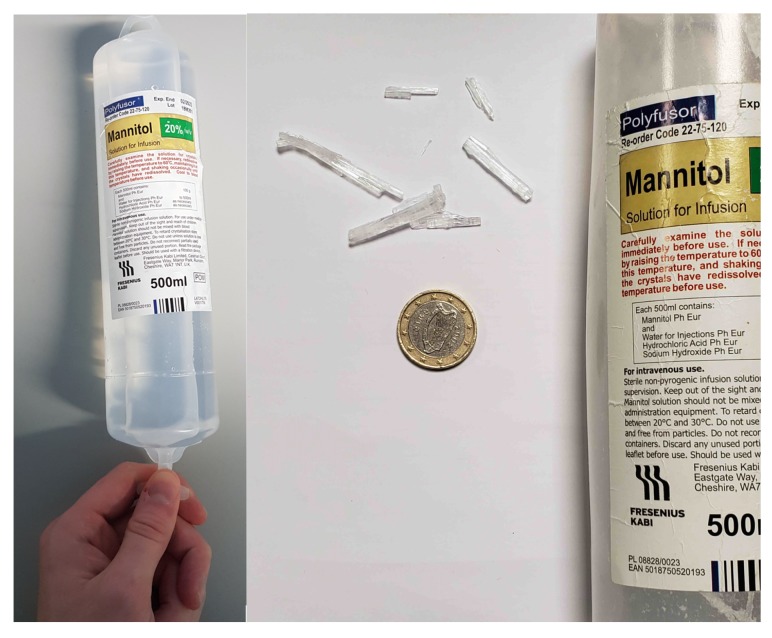
Photograph of mannitol infusion solution (**left**) and crystals were taken from solution after precipitation (**right**).

From a formulation perspective, the stability of mannitol infusion is unreliable as precipitation commonly occurs during storage of mannitol in concentrations >10% *w*/*v*, such as those employed in TBI. The current solution is to heat the mannitol infusions up to 60 °C (often in a sink or a bucket of hot water), wait for the crystals to dissolve and cool the solution back down to body temperature prior to administration. As intracranial hypertension is a medical emergency, this method is not rapid enough and carries a significant risk of patient harm. Further, in the ambient temperatures expected in clinical environments (15–25 °C), mannitol can precipitate during a procedure. Although risks can be reduced by using a filter at the end of the IV set, Lionel and Hrishi have noted the possibility of microparticulate embolism [12], not to mention the now unknown mannitol concentration. The instability of mannitol solutions is further highlighted by the red warning on the label reminding clinicians to, “carefully inspect the contents of the solution for crystals immediately before use.” (Figure 1). This places further emphasis on the need for new formulations of this important agent.

Mannitol solubility has been extensively studied for various pharmaceutical applications such that there is a comprehensive understanding of the thermodynamic characteristics of mannitol and the interplay between its various polymorphs in the liquid and solid states [13,14,15,16]. The purpose of this study is not to replicate this but to understand how to optimise this formulation, particularly as the properties of mannitol in a 20% *w*/*v* infusion solution within the clinical environment are not well understood.

Therefore, we set out to understand the stability limits of mannitol 20% *w*/*v* to create a safer, stable hyperosmotic formulation via combination with clinically acceptable osmotic agents (NaCl, sorbitol and glycerol [7,17]).

## 2. Materials and Methods 

### 2.1. Materials

All materials and reagents were purchased from Sigma-Aldrich at the highest purity available and used as received. Mannitol 20% *w*/*v* solution for infusion was donated as a gift from the University Hospital Limerick, purchased originally from Fresenius Kabi (batch number: 18M201, expiry: 02/2023).

### 2.2. Methods

#### 2.2.1. Powder X-ray Diffraction (PXRD)

X-ray powder diffraction patterns were collected in Bragg-Brentano geometry on an Analytical Empyrean diffractometer equipped with a sealed tube (Cu Kα12, λ = 1.5418 Å) and a 1D X’Celerator detector between 4 and 40 2𝜃 degrees.

#### 2.2.2. Differential Scanning Calorimetry (DSC) 

Thermogravimetric experiments were carried out using sealed aluminium pans on a Netzsch Polyma 214 differential scanning calorimeter. Temperature calibrations were made using indium as the standard. An empty pan, sealed in the same way as the sample, was used as a reference. All the thermograms were run at a heating/cooling rate of 20 °C·min^−1^ under a nitrogen purge at a rate of 50 mL·min^−1^.

#### 2.2.3. Cambridge Structural Database (CSD) Analysis

The Cambridge Structural Database (CSD; version 5.40, November 2019) was searched for all known mannitol solid forms. The calculated PXRD patterns for the various mannitol polymorphs were obtained and further analysis was conducted with the PANalytical X’Pert HighScore software package (version 4.7).

#### 2.2.4. Equilibrium Solubility Measurements

Solubility measurements of mannitol were performed by adding 3 g mannitol to 10 mL of degassed (via sonication), deionised H_2_O in 20 mL glass vials under constant stirring at 500 rpm. Solubility measurements were conducted at 7, 10, 15, 20 and 25 °C using temperature-controlled water baths. Once the solutions had been equilibrated for at least 24 h, a 2 mL sample was taken from the liquid phase and filtered (PTFE filters, 0.45 µm pores) into a pre-weighed glass vial and capped immediately. To avoid sampling errors, the syringes and filters were heated or cooled to the corresponding temperature. The mass of the sample vials was measured after cooling for 1 h. The vials were then uncapped and placed in a fume hood. Once evaporation was visibly complete, the vials were placed in the oven overnight and once completely dry the vials were weighed again. The effects of additives on mannitol solubility was determined as above in deionised H_2_O containing appropriate concentrations of additives. This procedure was repeated in triplicate.

#### 2.2.5. Continuous Cooling Experiments

All experiments were conducted in a 500 mL jacketed vessel (OPTIMAX 1001, Mettler Toledo, Columbus, OH, USA) with an overhead stirrer (250 rpm) and a temperature control system (accurate to 0.1 °C). An in situ Focused Beam Reflectance Measurement (FBRM) (Particle Track G400, Mettler Toledo, Columbus, OH, USA) probe was connected to a computer system to monitor the number of particles and their size distribution during the runs.

The reactor was filled with 500 mL 20% *w*/*v* mannitol solution and heated to 45 °C for 5 min to ensure complete dissolution of mannitol, the FBRM probe was calibrated in these conditions. Then the reactor solution was brought to 25 °C and the solution was subsequently cooled to 0 °C at 0.1 K·min^−1^. At the end of the experiment, the solution was brought back up to 45 °C for 5 min to dissolve the mannitol. This procedure was repeated in triplicate.

#### 2.2.6. Induction Time Experiments 

Six vials containing 20% *w*/*v* mannitol solution were prepared and complete dissolution of mannitol was obtained by heating (45 °C) and sonication for 5 min. All vials were then placed immediately into a temperature-controlled water bath (at 10, 15 and 18 °C) and a nucleation event was determined by placing a backlight into the bath and recording the time at which the first crystal was visible.

## 3. Results and Discussion

### 3.1. Characterisation of Crystals within Mannitol 20% w/v Infusion Solution

As received, the mannitol solution had large, visible crystal precipitates which were dissolved by placing in an ultrasonic bath at 45 °C for 5 min (Branson®, Frequency 40 kHz). The infusion bag was then placed onto a laboratory bench and, after one week, large crystals had again precipitated from solution. The infusion bag was then cut open to isolate the crystals for further analysis (Figure 1).

**Figure 2:**
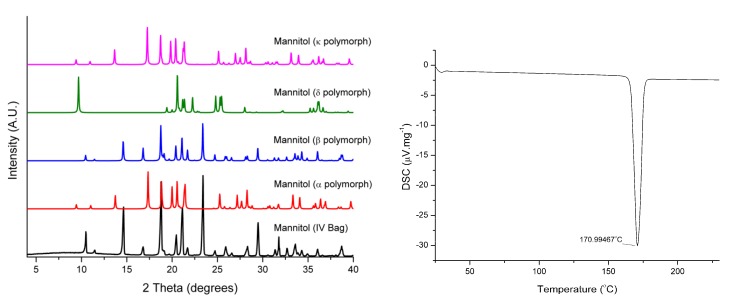
Powder X-Ray Diffraction (PXRD) (**left**) and Differential Scanning Calorimetry (DSC) (**right**) of mannitol crystals removed from the infusion solution.

To confirm the identity and purity of the precipitate, a number of the large needle-shaped crystals were pulverised and analysed by PXRD and DSC (Figure 2). The PXRD pattern in Figure 2 suggests that the crystalline form of mannitol found in the infusion solution is the 𝛽 polymorph, DSC confirms its purity. These results are unsurprising as the 𝛼 polymorph has been shown to nucleate from supersaturated solutions and then transform to the stable 𝛽 polymorph following Oswald’s rule of stages [18]. Although the existence of a mannitol hydrate has been documented in the literature, it is unstable [19]. This behaviour has been studied extensively for mannitol in aqueous systems [13,15,16,18].

### 3.2. Mannitol Solubility and Nucleation Characteristics in the 20% w/v Infusion Solution

**Figure 3:**
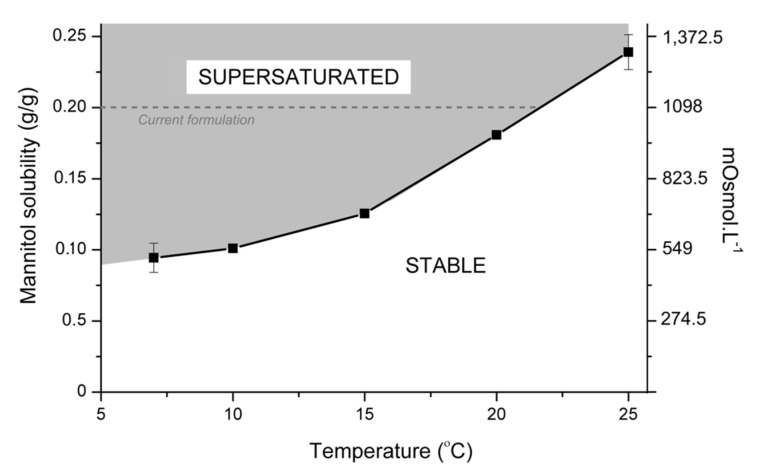
Solubility curve for mannitol in deionised water from 7 to 25 °C with the corresponding mOsmol·L^−1^ of mannitol solution at equilibrium calculated using Equation (1).

Equilibrium solubility experiments were conducted between 7 and 25 °C (Figure 3) to understand the stability of aqueous mannitol solutions across the range of temperatures they could be subjected to during transport and storage.

Extrapolating from the data presented in Figure 3, mannitol 20% *w*/*v* infusion will become supersaturated at ca. 21 °C and if stored at or below this temperature, crystallisation will be inevitable. This is in agreement with published literature [14,20]. As clinical environments are known to fluctuate between 15 and 25 °C there are moments when mannitol is stable and others where it is supersaturated and therefore capable of crystallising at any time. As temperatures drop further away from 21 °C supersaturation will increase, and so will nucleation rate [21]. Therefore, at the lower range of temperatures found clinically or expected in transit, mannitol instability would be expected to become increasingly difficult to manage. To the best of the authors’ knowledge, crystallisation kinetics of mannitol is not known under conditions that mimic the clinical environment. Crystallisation kinetics are dependent on the degree of supersaturation, influenced by the: temperature, concentration, viscosity, medium and agitation amongst others [21,22]. Understanding crystal nucleation kinetics will enable an appreciation of the rate of instability and this was investigated by induction time and continuous cooling crystallisation experiments [23].

To simulate the clinical environment, induction experiments were performed without stirring, as mannitol bags are commonly left unagitated in storage. The implications of this for crystal nucleation has been explored for the glycine system where the authors postulate that stirring can increase the nucleation propensity of metastable forms which can, in turn, induce the nucleation of stable forms [22], i.e., stirring could induce the nucleation of mannitol’s α polymorph which will transform to the stable β polymorph [18]. Stirring would falsely overestimate the instability issues of the mannitol infusion.

**Figure 4:**
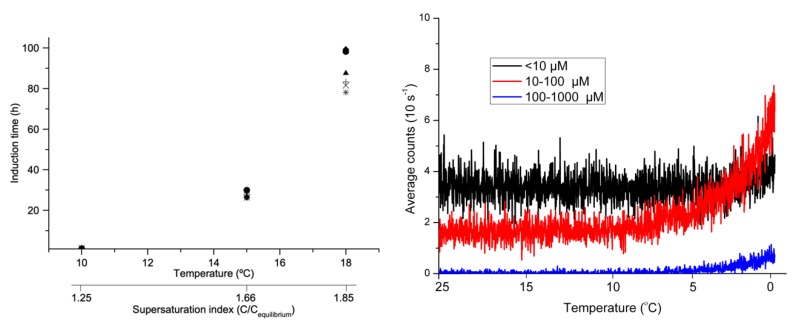
Induction time (h) at 10, 15 and 18 °C with corresponding supersaturation index (**left**) and continuous cooling experiments from 25 to 0 °C at 0.1 °C·min^−1^ (**right**).

Figure 4 reveals that complete nucleation occurs quickly at lower temperatures (e.g., <2 h at 10 °C) but can take several days at 18 °C. In continuous cooling experiments (Figure 4) nucleation occurs below 5 °C. This suggests that mannitol 20% *w*/*v* infusion has a large metastable zone, as it remains metastable from 21 to 5 °C. In previous experiments with higher cooling rates (2, 1, 0.5 and 0.25 °C·min^−1^), crystallisation was not observed beyond 0 °C (data not shown); it is known that higher cooling rates can extend the metastable zone [23]. In the clinical environment, temperature fluctuations, coupled with the large metastable zone and the long time for nucleation (ca. 100 h at 18 °C), could mean that in hotter climates, mannitol crystallisation is infrequently observed, if at all. This apparent ‘disappearance’ of crystallisation and therefore instability in these instances might explain the lack of attempts to reformulate a stable alternative. However, crystallisation could reappear when the right conditions are met [24], e.g., during winter or transport outside the hospital.

One intuitive solution is to reduce the mannitol concentration below the supersaturation concentration. However, as this approach reduces the osmotic potential of the infusion, it will, therefore, reduce its clinical effect. As such, reformulation must improve stability whilst maintaining clinical effectiveness. This could be achieved by combining mannitol with other hyperosmotic agents enabling stability and maintaining hyperosmolarity of 1098 mOsmol·L^−1^.

### 3.3. Mannitol Solubility in the Presence of Additives and Implications for Clinical Translation

Equilibrium solubility experiments were conducted in the presence of the various additives identified earlier in the literature review. This was to reveal the effect (if any) on mannitol solubility and identify suitable candidates for a new combination formulation.

**Figure 5:**
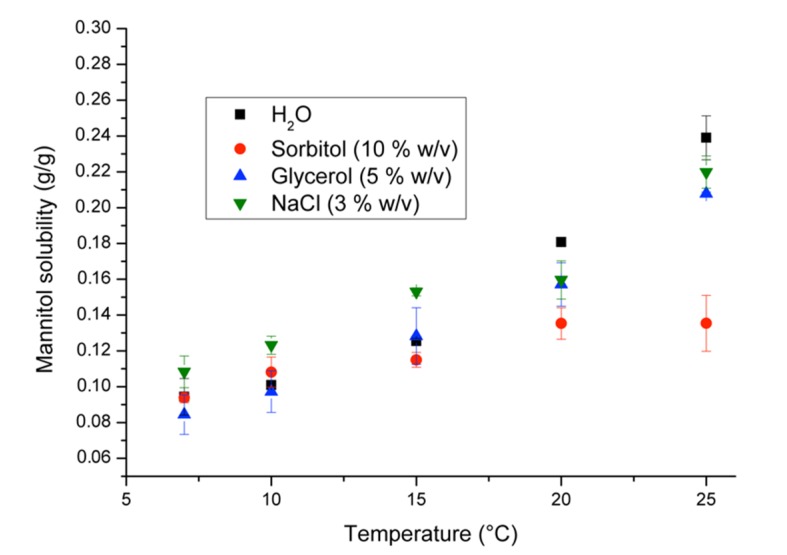
Solubility curve for mannitol in deionised water, glycerol (5% *w*/*v*), sorbitol (10% *w*/*v*) and NaCl (3% *w*/*v*) from 7 to 25 °C.

Figure 5 illustrates that the presence of sorbitol decreases the equilibrium solubility of mannitol significantly at 25 °C. This is possibly due to the increased viscosity of the solution conferred by sorbitol and its structural similarity to mannitol [25]. From a clinical perspective, the slight changes in solubility across the other additives will have a negligible effect on the stability of mannitol, i.e., mannitol 20% *w*/*v* solution will remain unstable below 21 °C. However, the presence of these additives could synergise with mannitol to enable new mannitol formulations which can achieve the same target mOsmol·L^−1^. Table 1 summarises how this could be achieved where osmotic concentration, commonly expressed as osmolarity (mOsmol·L^−1^) is determined by Equation (1), where 𝑛 represents the number of particles into which a molecule disassociates (mannitol = 1), which is determined for each species in the solution, 𝑖. Equation (2) expresses this calculation for the mannitol 20% *w*/*v* infusion.

(1) $$Osmolarity\;\left(mOsmol\cdot L^{-1}\right)=\;{\sum^{}}_{i}^{}molarity_{i}\times \;n_{i}\times 1000$$

(2) $$\frac{200\;g}{183\;moles}\times 1\times 1000=1098\;mOsmol\cdot L^{-1}$$

Each of the formulation additives studied could be utilised in a new, stable, hyperosmotic formulation and the amount of each component can be tuned as desired so long as mannitol concentration is below the equilibrium solubility (Table 1 and Figure 5). The choice of formulation additive will be determined by the clinical application as each additive has its own advantages and disadvantages. In vivo, sorbitol and glycerol are rapidly metabolised by the liver (therefore diminishing its osmotic effect) and may increase the risk of complications for patients with diabetes [17]. Mannitol is exclusively eliminated renally (indeed, this is a key feature of its therapeutic mechanism) and therefore is not suitable for patients with renal insufficiency [17]. With NaCl, associated side effects include myelinolysis and electrolyte imbalances [26]. In addition, the optimum administration protocol of NaCl is not well understood although research is ongoing [27].

## 4. Conclusions

This study fills an important gap in the literature describing the stability of 20% *w*/*v* mannitol infusion solution across the range of conditions it will be exposed to before reaching the patient. We have also evaluated a number of hyperosmolar alternatives which could be directly translated to the clinic. The advantage of these formulations is three-fold as they are tuneable, stable and therefore safer. Specifically, each formulation can be tuned by the pharmacist to suit the clinical application, eliminating the risk of recrystallisation in the controlled clinical environment (15–25 °C) whilst maintaining the target osmolarity and perhaps enabling synergistic mechanisms of action.

Future work would include an evaluation of the safety and efficacy of this formulation in vivo; particularly of interest would be a comparison of the efficacy of the mannitol/NaCl combination against either component alone. Indeed, perhaps the ongoing debate surrounding the choice of hyperosmolar therapy (salt vs. sugar [4]) reflects that both mannitol and NaCl have useful clinical effects in their own right. 

## Figures and Tables

**Table 1:** Formulation approaches to achieve the target of 1098 mOsmol·L^−1^.

Formulation (*w*/*v*)	Stable to (°C)	Mannitol mOsmol·L^−1^	Additive mOsmol·L^−1^
Mannitol (20%)	21	1098	-
Mannitol/Sorbitol (10%/10%)	10	549	549
Mannitol/Glycerol (10%/5%)	15	549	549
Mannitol/NaCl (7.54%/2%)	7	413.6	684.4
